# Supplementary material for: Are carbon emissions trading and green financial instruments synergistic? -Comprehensive quantitative research based on content analysis
Source: PLoS One. 2024 Mar 7;19(3):e0298601. doi: 10.1371/journal.pone.0298601 (PMC10919601; doi:10.1371/journal.pone.0298601)
Supplement: S1 Data — (ZIP) [file pone.0298601.s001.zip › Data/地级市Z_F工作报告.zip!/╘╞─╧/╟·╛╕╩╨/└┤╫╘╣1⁄2╓┌║┼í╛┬φ┐╦╩2╛▌═°í┐_2018.docx]

各位代表：

我代表市人民政府，向大会报告工作，请予审议，并请市政协委员和列席人员提出意见。

一、过去五年的工作回顾

市四届人大一次会议以来的五年，曲靖走过了极不平凡的奋斗历程。五年来，面对经济下行压力加大、结构调整阵痛加剧、矛盾风险集中显现等严峻复杂形势，我们在省委、省政府和市委的坚强领导下，全面贯彻落实党的十八大、十九大精神和习近平总书记考察云南重要讲话精神，坚定不移贯彻新发展理念，积极应对各种矛盾风险挑战，统筹抓好改革发展稳定各项工作，全面建成小康社会迈出了坚实步伐。

——全力以赴稳增长促发展，经济实现企稳回升。面对2014年经济断崖式下滑、连续两年没有完成目标任务的严峻形势，面对煤矿停产整顿、房地产市场调整、烟草产业计划调减等诸多困难，出台了扩大社会投资、降低企业成本、扩销促产增效等一系列有力措施，完善经济运行监测调度机制，持续开展“狠抓落实年”工作，推动了经济止跌回升、恢复性增长。市内生产总值、规模以上工业增加值年均分别增长8.1%、7.5%，一般公共预算收入是2012年的1.3倍。固定资产投资、社会消费品零售总额年均分别增长22.2%、13.1%。城乡常住居民人均可支配收入年均分别增长9.3%、11.7%，均高于经济增长速度。

——扎实推进供给侧结构性改革，转型升级取得积极成效。作为全省去产能任务最重的州市，我们提高政治站位，顶住压力、克服困难，坚定不移实施“三去一降一补”。关闭煤矿矿井221对、退出煤炭产能1759万吨，取缔“地条钢”产能152万吨，淘汰工业落后产能407.7万吨，商品房去库存1105万平方米，为企业降低电费49亿元，减免退税109.5亿元。面对新旧动能转换缓慢的困境，坚持稳住存量与做大增量并举，积极构建现代产业体系。确定重点发展6大产业，制定实施意见、发展规划和五年行动计划，狠抓招商引资和项目落地，推动了高原特色农业蓬勃发展、工业经济逆势上扬、服务业快速增长，三次产业结构比由2012年的20:43.8:36.2调整为18.1:39:42.9。

——全力加快基础设施建设，城乡发展空间不断拓展。全力打好“五网”基础设施“三年攻坚、五年会战”，完成投资2055亿元。沪昆高铁建成通车，曲靖迈入高铁时代；新增高速公路通车里程541公里，率先在全省“县县通高速”，实现所有建制村通硬化路，被列为全国性综合交通枢纽和区域级流通节点城市。阿岗、车马碧水库开工建设，建成骨干水源工程19件，新增蓄水库容1.4亿立方米。新建、改造输配电线路6790千米；“气化曲靖”建设扎实推进，建成天然气输气管道678.9公里。实现光纤宽带、4G网络行政村全覆盖。统筹推进绕城高速公路围合区内673平方公里开发建设，沾益成功撤县设区，麒沾马百万人口大城市初具雏形。实现城市基础设施建设投资983.5亿元，完成15个乡镇改设33个街道区划调整工作，县以上建成区面积达257.6平方公里。智慧工商、智慧养老等项目建设成效明显。

——全面打好脱贫攻坚战，农村经济稳步发展。把脱贫攻坚作为重大政治任务，层层压实脱贫攻坚责任，五级单位真帮实扶，万名干部驻村入户，专项扶贫、行业扶贫、社会扶贫“三位一体”大扶贫格局全面形成。精准识别、精准帮扶、精准管理扶贫对象，脱贫攻坚政策框架基本形成、体制机制逐步健全。累计投入各类扶贫资金462亿元，建档立卡贫困人口减少80.34万人，贫困发生率下降11.2个百分点，贫困地区农民人均可支配收入增加5866元，贫困地区生产生活条件明显改善。现代农业稳步发展，粮食产量稳居全省第一，8个县（市、区）被评为产粮大县。在全省率先开展“百千万”畜禽示范场和“牛源体系”建设，油菜、魔芋、蚕桑等高原特色农业快速发展。培育农业龙头企业502个、家庭农场1930户、种植大户3763户，建成涉农院士专家工作站25个，新增国家驰名商标农产品4个、认证“三品一标”农产品40个。

——深入推进改革开放，城市竞争力明显提升。制定并公布市、县、乡三级权责清单，建成行政审批网络服务大厅和投资审批中介超市，“放管服”、营改增、农村水利、事业单位等重点领域改革成效明显。国家循环经济示范市、食品安全示范城市、森林城市和公共文化服务体系示范区创建进展顺利，国家新型城镇化综合试点通过终期评估，养老服务业综合改革试点基本完成，获批创建全国质量强市示范城市。成功举办国际蓝莓大会、中国液态金属高峰论坛、硅晶材料论坛。成功举办世界铁人三项赛、第14届省运会等一系列国际国内赛事，开通“曲靖号”文化旅游专列，“曲靖舰”入列南海舰队，与深圳市、金华市等友好城市交流合作进一步加强。扎实推进“四城联创”工作，成功创建国家卫生县城3个、国家园林县城1个，荣获“国家卫生城市”“国家园林城市”荣誉称号，城乡人居环境明显提升。

——全面发展社会事业，人民群众获得感明显增强。着力解决人民群众最关心最直接最现实的利益问题，财政支出的76%以上用于保障和改善民生。学前三年毛入园率、义务教育巩固率和高中阶段教育毛入学率分别达78%、94.3%、87.6%，麒麟、沾益、马龙、罗平、师宗通过国家义务教育基本均衡发展评估认定。在全省率先实施药品采购“两票制”改革，全部取消公立医院药品加成。建成三级甲等医院6所。兑现大病保险补偿费用3.5亿元。市文化体育公园全面建成投入使用。覆盖城乡居民的社会保障体系基本建立，社会救助体系逐步完善，发放救助资金58.5亿元。经受住了“8·03”地震等自然灾害的考验，会泽灾区灾后恢复重建全面完成。

——切实加强生态文明建设，城乡环境明显改善。建立中央环保督察反馈意见整改落实工作台账，制定联席会议、督察督办、通报考核、销号管理等工作制度，解决了一批历史遗留问题。深入实施大气、水、土壤污染防治行动计划，实施重点减排项目980个、中小河流治理工程22件、重金属污染防治项目11个，单位生产总值能耗累计下降20.8%。划定基本农田保护面积882万亩，改造中低产田地215.6万亩，土地矿产资源节约集约利用水平不断提升。深入推进城乡绿化攻坚行动，完成营造林357.4万亩，森林覆盖率提高5个百分点。建成国家级生态乡镇4个，实施农村环境综合整治项目362个，县城以上“两污”设施实现全覆盖。

——落实全面从严治党要求，政府治理能力进一步提高。扎实开展党的群众路线教育实践活动、“三严三实”和“忠诚干净担当”专题教育，推进“两学一做”学习教育常态化制度化。出台加强政府党组自身建设的意见，全面整改巡视巡察反馈问题。认真执行市人大及其常委会的决议决定，自觉接受市人大的法律监督、工作监督，主动接受市政协的民主监督，办理答复人大代表议案、建议765件，政协提案1551件。深入推进政务公开，全面落实法律顾问制度，实行重大行政决策专家咨询论证，法治政府建设通过评估。公共资源交易总额837.9亿元，节支18.6亿元。审计查出并纠正违纪违规资金16.8亿元。驰而不息纠正“四风”，查处违反中央八项规定精神典型案件96起，政纪处分32人。深刻汲取“4·07”“4·21”煤矿事故教训，采取超常规措施强化安全监管和基础能力建设，连续三年未发生重特大安全生产事故。化解了一批矛盾纠纷和信访积案，立体化社会治安防控体系基本建成。

各位代表！刚刚过去的2017年，我们坚持稳中求进工作总基调，以供给侧结构性改革为主线，统筹抓好稳增长、促改革、调结构、惠民生、防风险各项工作，全面完成了市四届人大六次会议确定的目标任务。实现市内生产总值1941.1亿元，增长10%；固定资产投资2244.8亿元，增长25.1%；社会消费品零售总额635.3亿元，增长12.5%；一般公共预算收入136.2亿元，增长7.8%；城乡常住居民人均可支配收入分别为31932元、11345元，分别增长8.3%、9.3%；单位生产总值能耗下降3.1%；居民消费价格指数上涨0.6%；城镇登记失业率为2.6%。

一年来，我们重点抓了以下工作：

（一）全力推动经济平稳较快增长。出台稳增长36条政策措施，定期听取县（市、区）重点工作推进情况汇报，新增银行贷款84亿元，推动13户重点工业企业全面复产。全社会用电量达260.3亿千瓦时，增长15%。落实重点项目包保责任制、定期观摩制、现场办公制，争取上级各类补助资金275亿元，完成产业投资880亿元，69个重点产业项目竣工投产，增添了经济发展动力。宣威、陆良分别进入全省县域经济发展10强县和跨越发展先进县。完成园区基础设施投资40亿元。民营经济增加值占市内生产总值的比重达49.5%。

（二）全力加快重点产业发展。种植特色经济作物538万亩，创建“百千万”畜禽示范场27个，新增规模以上农业龙头企业10个，蓝莓工程技术研发中心等项目建成投产，温氏集团生猪养殖等项目进展顺利，第一产业增加值增长6%。今飞500万件铝合金摩托车轮毂一期等项目建成投产，万里扬300万台汽车变速器零部件等项目加快推进，阳光能源硅材料等项目开工建设；完成一汽红塔国V系列轻卡生产线改造，蓝舰T340皮卡正式投产；众一化工5000吨H酸、宣威恒邦40万吨钙镁磷肥技改等项目进展顺利，规模以上工业增加值增长11%。出台流通节点专项规划、现代商贸物流产业发展规划，万达广场等269个项目建成运营；全域旅游示范区创建扎实开展，珠江源旅游区等31个项目稳步推进，规模以上文化企业达33户，国际高原体育城建设成效明显，第三产业增加值增长10%。认定国家高新技术企业18户、省级科技型中小企业98户，新增市级以上院士专家工作站48个、云南名牌15个。

（三）全力推进精准扶贫精准脱贫。深入开展“找问题、补短板、促攻坚”等专项行动，各项脱贫举措精准落实到户到人。整合各类资金210.8亿元，扶贫资金投入力度前所未有。实施易地扶贫搬迁2.9万人、农村危房改造4.56万户，发展特色产业带动32.8万贫困人口增收，转移就业、健康、教育、社保等扶贫措施全面落实，会泽、宣威等深度贫困地区脱贫攻坚工作扎实开展，可望完成“罗平县摘帽，12个贫困乡、97个贫困村出列，11.25万贫困人口脱贫”的年度目标任务。

（四）全力加强城乡基础设施建设和生态环境保护。西河公园等40个项目全面建成，麒马大道等38个项目启动建设，麒沾马一体化步伐加快。改造棚户区3879套。宣曲等5条高速公路建成通车。渝昆高铁等项目前期工作扎实开展。宣威支线机场和会泽、罗平、沾益通用机场前期工作有序推进。阿岗水库成功截流，车马碧水库建设快速推进。建成35千伏及以上输变电工程14个，中缅天然气管道昭通支线曲靖段、陆良支线一期贯通。光纤宽带和4G通信网络加速向自然村延伸。划定生态保护红线，制定《会泽黑颈鹤国家级自然保护区管理办法》，中央环保督察反馈问题扎实整改。启动南盘江综合治理工程，建成城市污水管网112公里，所有河湖库渠实现河长全覆盖。市危险废物集中处置项目建成投入运行，会泽者海土壤污染修复治理等项目稳步推进。完成营造林75.1万亩，森林覆盖率达47%。城乡人居环境提升专项整治成效显著，麒麟、马龙创建为省级生态文明县。

（五）全力深化改革扩大开放。取消行政审批事项12项、其他行政权力事项23项，清理规范行政审批中介服务事项85项。制定“放管服”改革9条措施，实施“50证合一”改革，商事制度改革被国务院通报表扬，国家工商总局曲靖商标受理窗口开通。承担行政职能事业单位改革试点顺利完成。参加《魅力中国城》竞演，获评“优秀魅力城市”。第十一届国际园博会“曲靖园”建成对外开放，与俄罗斯伏尔加格勒市、泰国佛统市签订友好城市意向书。到位市外国内资金988亿元，完成外贸进出口总额8.7亿美元。

（六）全力保障和改善社会民生。新建普惠性幼儿园87所，曲靖一中麒麟学校等5所学校建成投入使用，完成“全面改薄”203所。新增义务教育学位2.9万个，消除了入学新生“大班额”现象。完成曲靖医专新校区选址，启动曲靖职业技术学院筹建工作。创建学联体3个、“八优”学校93所。市中医院新院等项目稳步推进，建成3个特色专科诊疗中心一期项目。分级诊疗制度深入推进，组建市县医疗联合体60个。建成殡仪馆6所、乡镇公益性公墓123个。发放创业贷款15.6亿元，扶持创业1.5万人，培育“两个10万元”微型企业1300户。城乡居民医疗保险制度全面整合，财政补助标准提高到450元。市养老综合服务中心投入使用，市老年公寓一期即将运营，建成公办和民办养老机构26个。群众性文化体育活动蓬勃发展，竞技体育水平进一步提高。26个民族团结进步示范点建设加快推进。建立安全生产巡查制度，全面建成煤矿安全生产信息化平台，深入开展专项整治和打非治违专项行动，事故起数、死亡人数、直接经济损失同比分别下降3.7%、5.4%、58.9%。建成出入境检验检疫曲靖技术分中心、食品药品检验检测联合实验室。深入推进“智慧公安”建设，推行村（社区）网格化服务管理，人民群众安全感进一步提升。

全力支持各民主党派、工商联、无党派人士参政议政，全力支持工会、共青团、妇联等人民团体和社会组织发挥作用。国防动员和后备力量建设稳步推进，军转安置、拥军优抚、双拥共建工作成绩突出。国家安全、广播电视、社会科学、外事、侨务、档案、保密、史志、地震、气象、移民、水文、邮政、统计和统计调查等工作取得新成绩，妇女、儿童、青少年、残疾人、慈善等事业取得新进步。

各位代表！五年的奋斗历程启示我们：必须以习近平新时代中国特色社会主义思想为指导，自觉维护以习近平同志为核心的党中央权威和集中统一领导，让各项工作始终沿着正确的方向前进；必须坚持全面从严治党，切实抓好政府自身建设，大力弘扬“跨越发展、争创一流，比学赶超、奋勇争先”精神，确保党中央、国务院和省委、省政府各项决策部署在曲靖落地生根；必须坚持发展是解决一切问题的基础和关键，在全力赶超中加快转型、在转型升级中谋求跨越，走出一条符合曲靖实际的发展路子；必须坚持问题导向，以改革促进开放、以开放倒逼改革，破除一切不合时宜的思想观念和体制机制弊端，为经济社会发展注入持久动力；必须坚持发展为了人民、发展依靠人民、发展成果由人民共享，努力满足人民对美好生活的向往。

各位代表！以上成绩和经验的取得，是省委、省政府和市委坚强领导、科学决策的结果，是市人大、市政协有力监督、倾力支持的结果，是全市广大干部群众同心协力、艰苦奋斗的结果。在此，我代表市人民政府，向全市广大干部群众，向人大代表、政协委员、各民主党派、工商联和无党派人士、各人民团体、离退休老干部、社会各界人士，向驻曲人民解放军、武警官兵和中央、省属单位，向为曲靖经济社会发展挥洒汗水、辛勤努力的历任政府领导班子，向所有关心支持曲靖改革发展的同志们，致以崇高的敬意，并表示衷心的感谢！

在充分肯定成绩的同时，我们也清醒地认识到存在的问题和面临的挑战。主要是：脱贫攻坚时间紧、任务重，脱贫举措还不够精准，与下足“绣花”功夫的要求差距大；实体经济面临困难较多，经济质量和效益不高，与高质量跨越式发展的要求差距大；科技创新能力不强，传统产业转型升级缓慢，新兴产业发展不足，与构建现代化产业体系的要求差距大；城乡区域发展不平衡不充分，生态环境保护任重道远，与城乡融合发展的要求差距大；优质教育、医疗卫生资源不足，公共服务均等化水平不高，与人民对美好生活的向往差距大；安全生产基层基础薄弱，治安防控体系还不完善，与社会治理社会化、法治化、智能化、专业化的要求差距大；政府职能转变和干部作风建设有待加强，营商环境还需进一步优化，与建设人民满意政府的要求差距大。针对这些问题，我们一定高度重视，采取有效措施加以解决。

二、今后五年的主要任务

各位代表，今后五年，是全面脱贫、全面建成小康社会的决胜期，也是“两个一百年”奋斗目标的历史交汇期。我们必须高举中国特色社会主义伟大旗帜，以习近平新时代中国特色社会主义思想为指导，紧紧围绕党的十九大确定的战略目标，统筹推进“五位一体”总体布局，协调推进“四个全面”战略布局，坚持稳中求进工作总基调，贯彻落实新发展理念，紧扣社会主要矛盾变化，深入推进供给侧结构性改革，增强经济发展质量优势，推动新型工业化、信息化、城镇化和农业现代化同步发展，全面开启建设现代化经济强市新征程。

各位代表，新时代赋予新使命，新使命要有新作为。未来五年，结合市第五次党代会精神和“十三五”规划目标，按照市委五届四次全会的部署，提出以下奋斗目标：

——到2020年，全面打赢防范化解重大风险、精准脱贫、污染防治三大攻坚战，乡村振兴取得重要进展，产业结构调整迈出决定性步伐，全面建成小康社会，进入全国地级市100强。

——到2022年，基本建成可支撑未来发展的现代化经济体系，力争6大重点产业增加值占市内生产总值的比重达65%，市内生产总值年均增长10%以上，建成云南经济强市、对内对外开放的重要枢纽城市、特色生态文化旅游城市。

各位代表，实现上述目标，必须发扬时不我待、只争朝夕的精神，不驰于空想、不骛于虚声，奋力实现更高质量、更有效率、更可持续的发展，努力让人民群众更有获得感幸福感安全感。

——坚决打赢精准脱贫攻坚战。紧盯43.46万贫困人口，聚焦深度贫困地区，全力奋战1000天，坚决兑现“小康路上不让一个贫困群众掉队”的庄严承诺。紧盯绝对贫困人口，聚焦“两不愁、三保障”，既不吊高胃口、也不降低标准，聚合各项政策、统筹各类资金，下足“绣花”功夫，力戒形式主义，构建贫困人口稳定脱贫长效机制。抓住产业扶贫根本，通过股份合作、企业带动等形式，确保有劳动能力的贫困人口产业扶贫全覆盖。落实易地扶贫搬迁政策，推动贫困人口向城镇搬迁，确保搬得出、稳得住、能致富。统筹推进生态、教育、健康、转移就业、社会救助兜底保障等脱贫举措，确保脱真贫、真脱贫。

——加快云南经济强市建设。推动新型工业化和信息化融合发展。以争创“中国制造2025”国家级示范区为契机，瞄准国际国内领先水平，集中力量发展先进制造业和战略性新兴产业，着力振兴实体经济，努力建设制造业强市。实施传统产业提质增效和新兴产业培育工程，充分发挥云南绿色能源优势和曲靖良好的工业基础优势，推动有色金属深加工及液态金属、精细化工、军民融合现代装备制造、生物资源加工等产业集群发展。大力发展“互联网+”产业，推动互联网、大数据、人工智能和实体经济深度融合，全面建成“智慧曲靖”。坚持新型城镇化与乡村振兴战略同步推进。以珠江源大城市为核心、县城为重点、特色小城镇为依托、美丽宜居乡村为基础，推动城乡区域协调发展，城镇化率年均提高1个百分点以上。聚焦麒沾马片区，坚持精品化建设、园艺化打造、智慧化管理，推动政策制度衔接联动、基础设施互联互通、公共服务共建共享，着力发展高端制造、科技创新、总部经济、现代商圈、金融商务、文化创意等新兴产业，建立多元化房地产供应体系，打造功能完善、产业兴旺、充满活力、宜居宜业、独具魅力的云南第二大城市。充分发挥曲靖作为全省第一农业大市的优势，加快构建高原特色现代农业产业体系、生产体系和经营体系，打好绿色食品这张王牌。坚持土地适度规模经营，推行“大产业+新主体+新平台”的产业发展模式，建成一批农业产业化联合体、田园综合体、农民合作示范社、家庭农场，推进农村一二三产业融合发展。坚持科学规划、注重质量、从容建设，建立健全城乡融合发展体制机制和政策体系，加强农村基础设施、公共服务、生态环境、人才队伍建设，构建新型工农城乡关系。传承发展提升农耕文明，健全自治、法治、德治相结合的乡村治理体系。提高全要素生产率。推进创新型城市建设，力争重点产业科技领军人才、院士专家工作站、重点实验室、工程技术研究中心全覆盖。弘扬工匠精神，建立技术支撑、综合监管、社会信用、责任追溯体系，创建全国质量强市示范城市。优化金融发展环境，增强金融服务实体经济能力。推进“珠源”系列人才和企业家培养工程，建设知识型、技能型、创新型劳动者大军，将人力资源优势转化为人才优势。

——加快对内对外开放的重要枢纽城市建设。打造互联互通的基础设施网络。抓住全国性综合交通枢纽城市建设机遇，加快交通连接通道和重要节点建设，构建客运“零距离换乘”、货运“无缝衔接”的交通运输网络体系。增加路网密度，新增高速公路295公里以上，新建和改建农村公路5000公里以上；积极支持渝昆高铁、南昆铁路扩能改造、师宗至文山铁路建设；建成宣威支线机场、3个通用机场、若干个水上机场。坚持物流枢纽、物流园区、专业市场与综合交通体系同步建设，建成全国区域级流通节点城市。建成阿岗、车马碧水库，开工建设黑滩河大型水库和3件中型水库，新增蓄水库容3亿立方米，基本建成供水安全保障网。加快城乡电网改造升级，全面完成“气化曲靖”建设任务。打造支撑有力的发展平台。坚持招商引资与招商引智相结合，建设一批产业活力强劲、服务功能完备的工业园区，力争园区工业总产值占全市工业总产值的比重达80%以上，将工业园区打造成产业发展的平台、对外开放的窗口。打造服务高效的营商环境。深化行政管理体制改革，推行“互联网+政务服务”，推动行政审批标准化，建立以服务企业为中心的体制机制。全面实施市场准入负面清单制度，推动“双随机、一公开”全覆盖，更好激发市场主体活力。优化政策环境、市场环境、法治环境，推动民营经济加快发展。建设对内对外开放新高地。主动融入国家和省发展战略，加速融入滇中城市经济圈发展，加强与周边地区的经济协作，深化友好城市间的务实合作，增强城市辐射力和影响力。发挥招商分局和商会、行业协会作用，着力引进、培育一批国际贸易企业，积极推进综合保税区建设。

——加快特色生态文化旅游城市建设。认真落实全省打造“健康生活目的地”工作部署，统筹推进生态文明建设和文化、体育、旅游产业发展，大力提升城市美誉度和影响力。打造珠江绿源。严守生态红线，全面落实主体功能区规划，开展国土绿化行动，深入实施城乡人居环境提升工程，推进生态文明市、森林城市、循环经济示范市创建，厚植生态底色，让曲靖的天更蓝、水更清、山更绿、空气更清新。发展生态产业。围绕“大健康+生态建设”主题，推广林上产品加工、林中观光旅游、林下种养结合的立体经营模式，推动生态健康产业发展，把生态优势转化为发展优势。推动文体旅融合发展。强化本土文化发掘，提升城市文化内涵，打造文化品牌。加强文化遗产保护利用，培育一批骨干文化企业，繁荣文化市场，让文化软实力成为曲靖发展的硬支撑。建设一批综合性全民健身产业园，深入开展群众性文化体育活动，打造一批国际高原体育赛事品牌，提高竞技体育水平，全面建成国际高原体育城。把麒沾马作为全市最大的景区、最美的旅游目的地来建设和经营，统筹全市全域旅游和重点景区、特色小镇建设，打造钻石线路，优化配套服务，打响“大珠江源”旅游品牌，将旅游业发展成为全市重要的支柱产业。

——不断提高人民群众获得感幸福感安全感。建设区域教育中心。加快普惠性幼儿园建设，推动义务教育优质均衡发展，大力发展高中教育、民族教育、特殊教育，基本普及十五年教育。支持曲靖师范学院创建综合性大学、曲靖医专和云南能源职业技术学院升本，建成曲靖职业技术学院，争取知名高校到曲靖建设分校或研究院。建设区域医疗卫生中心。深化医药卫生体制改革，全面推进市县医疗联合体、县乡村医疗共同体建设，提升医疗卫生服务能力，基本建立分级诊疗服务体系。大力发展健康产业，改善健康环境，建设健康曲靖。扎实开展国家文明城市创建。实施文艺精品工程、文化惠民工程、传统文化传承工程，讲好曲靖故事、传播曲靖声音。推进公共文化体育服务标准化、均等化，建成国家公共文化服务体系示范区。建立健全多层次社会保障体系，实施更加积极的就业创业政策，城镇登记失业率控制在4.3%以内。压实安全生产责任，深化信访工作制度改革，完善社会治安防控体系，提高社会治理社会化、法治化、智能化、专业化水平。

三、奋力实现高质量跨越式发展新开局

2018年，是贯彻落实党的十九大精神的开局之年，是改革开放40周年，是决胜全面建成小康社会、实施“十三五”规划承上启下的关键一年，也是新一届政府履职的第一年。我们必须以习近平新时代中国特色社会主义思想为指导，全面贯彻落实党的十九大精神、中央经济工作会议精神、习近平总书记考察云南重要讲话精神，按照省委、省政府和市委的决策部署，坚持稳中求进工作总基调，以深化供给侧结构性改革为主线，坚持“四化同步”，统筹抓好稳增长、促改革、调结构、惠民生、防风险各项工作，持续开展“狠抓落实年”工作，坚决打好产业发展、脱贫攻坚、城乡融合“三大会战”，在工业转型发展、基础设施建设、生态文明建设、改革开放、民生改善、社会治理等方面取得重大进展，保持经济社会大局稳定，推动全面从严治党实现新突破，奋力谱写新时代曲靖高质量跨越式发展新篇章。

经济社会发展的主要预期目标建议为：市内生产总值增长9.5%，规模以上固定资产投资增长20%，规模以上工业增加值增长10%，社会消费品零售总额增长12%，一般公共预算收入增长7%，城乡常住居民人均可支配收入分别增长8.5%、9.5%，单位生产总值能耗控制在省下达目标以内，城镇登记失业率控制在3.3%以内，师宗、富源脱贫摘帽，312个贫困村出列，16.3万贫困人口脱贫，会泽、宣威等深度贫困地区脱贫攻坚取得决定性进展。

实现上述目标，将重点抓好以下8项工作：

（一）集全社会之力坚决打好精准脱贫攻坚战

压实脱贫攻坚责任。围绕2020年全面打赢脱贫攻坚战目标，算清时间账、任务账，增强责任感、紧迫感，提升攻坚能力，强化监督考核，提高脱贫质量。以更大的决心、更清晰的思路、更精准的举措，举全市之力、集全市之智，攻坚拔寨、全力冲刺，确保全年目标任务圆满完成。

全面落实精准扶贫举措。打好易地扶贫搬迁攻坚战，用好国家城乡建设用地增减挂钩等政策，完成易地扶贫搬迁41735人，推动搬迁人口100%向城镇集中安置。打好产业扶贫攻坚战，扶持专业合作社650个，建设特色产业基地380万亩，确保产业和新型农村合作经济组织对贫困户全覆盖。打好生态扶贫攻坚战，实施新一轮退耕还林20.7万亩、天保公益林建设3.9万亩，发展林下经济2万亩，带动1.5万贫困人口稳定脱贫。打好健康扶贫攻坚战，认真落实健康扶贫30条措施，切实减少因病致贫返贫。打好教育扶贫攻坚战，保障建档立卡贫困家庭学生资助全覆盖，惠及12.2万贫困学生。打好贫困群众素质提升攻坚战，对建档立卡贫困劳动力实施技能培训全覆盖，新增贫困劳动力转移就业2.75万人。打好农村危房改造攻坚战，实施“4类重点对象”农村危房改造7.2万户。打好贫困村脱贫振兴攻坚战，推动贫困村综合服务场地建设，发展贫困村集体经济，惠及25.5万贫困群众。织牢民生保障“兜底网”，全面实现扶贫线和低保线“两线合一”。

创新精准扶贫方式。安排市级专项扶贫资金6亿元以上，整合各类项目资金80亿元以上，争取金融扶贫资金50亿元以上、社会帮扶资金4亿元以上，重点扶持贫困地区产业发展。加大沪滇扶贫协作力度，开展“百企帮百村”和“互联网+”精准扶贫行动，引导社会力量广泛参与扶贫。扎实开展“自强、诚信、感恩”主题教育，防止政策“养懒汉”，增强贫困群众脱贫的内生动力。实施扶贫资金全程在线监控管理，全面实行项目资金公示公告制。开展扶贫领域腐败和作风问题专项治理，坚决防止虚假脱贫、数字脱贫。加强驻村工作队管理和扶贫队伍培训。

（二）以工业突破带动经济加速发展

加快传统产业转型升级步伐。开展烟草产业全国知名品牌示范区创建，拓展卷烟市场，发展卷烟配套产业，巩固“两烟”在全国的市场份额。深化煤炭产业供给侧结构性改革，建立煤炭市场精准调控机制，完成转型升级建设项目初步设计审批工作，稳妥处置涉煤利益冲突。稳定发展精细化工产业，推动钢铁行业规范条件公告尽快获批，引进一批下游深加工项目，为转型发展赢得空间。

推动产业集群发展。着力发展硅材及硅电子产业，推动云芯硅材扩能技改、阳光能源硅材料、晶龙电子材料等项目加快建设，力争洛阳中硅、西安华晶、河南恒星等项目开工建设。着力发展铝基新材料产业，力争今飞500万件汽车轮毂、500万件摩托车轮毂全部达产，万里扬300万台汽车变速器零部件等项目建成投产，推动大长江摩托车零配件、60万吨阳极碳素等项目尽快开工建设。着力发展液态金属产业，打通科技研发、成果转化、市场应用通道，重点推进液态金属电子电路、芯片散热器等项目建设，加快液态金属质量标准和检测检验体系建设。着力发展军民融合现代装备制造业，举办军民融合产业发展论坛，争取新能源汽车项目落地，推动动力电池、电机等配套产业发展，力争一批电子信息产业项目开工建设。推动昇兴罐装项目尽快开工建设，引进上下游项目集群发展。

构建科技创新、现代金融、人力资源与实体经济协同发展机制。积极争创“中国制造2025”国家级示范区，启动国家级高新技术产业开发区、曲靖经开区高新技术产业园区建设和全国质量强市示范城市创建。加强重点产业技术支撑体系和能力建设，组建产业技术研究院，争取省支持建设铝工业工程技术研究中心、硅工业工程技术研究中心。新增国家高新技术企业10户、省级科技型中小企业30户、市级以上院士专家工作站5个。健全信息共享、银担合作、财税激励等机制，增强金融对实体经济的支撑能力。建设创新型人才服务中心，采取“候鸟型”等方式引进人才，建设一批众创空间、星创天地。组建工业人才智库，加强本地企业家培养，采取订单式、学徒制等方式培养技能人才。推动园区集约发展，确保所有污水集中处理设施全面建成，完成园区基础设施投资50亿元，园区工业总产值占全市工业总产值的比重提高到74%。

抓实精准招商和项目落地。建立市县联动机制，压实招商责任，组建川渝招商分局，强化商会建设，制定招商图谱，大力开展产业链招商，策划包装重点招商项目100个以上，确保完成招商引资年度目标任务，项目履约率达70%以上，产业项目到位资金占实际到位资金的50%以上。狠抓项目落地，每月召开1次重点产业推进和现场办公会，督促每个县（市、区）各抓实10个产值上亿元的产业项目，力争产业投资占固定资产投资的比重提高2个百分点，制造业投资占产业投资的比重达33%以上。落实停止征收坝区耕地质量补偿费等降成本措施，进一步优化营商环境。落实支持民营经济创新发展政策措施，力争民间投资占固定资产投资的比重提高2个百分点，民营经济增加值占市内生产总值的比重提高到50%。

（三）以城市经济快速发展带动城乡融合发展

加快发展城市经济。坚持以产兴城、产城互动，以麒沾马为核心，壮大城市产业、完善城市功能、增强城市活力，吸引更多的外来人口到曲靖休闲度假、置业、创业，形成城市经济发展的主引擎。大力发展高端制造业，推动科技研发、仓储物流、信息服务、金融资本、人才培养等生产性服务业发展。加快发展休闲购物、教育培训、康体养生、文化娱乐、餐饮美食、家政服务等生活性服务业，推动城市农业与文化、旅游、体育产业融合发展。紧扣“特色、产业、生态、易达、宜居、智慧、成网”七大要素，高质量、高标准推进特色小镇建设。引导房地产企业走差异化、品质化的路子。

着力提高城市品位。继续抓好全国文明城市创建工作。开展城市设计、“城市双修”和国家新一轮土地利用总体规划编制试点，推进“多规合一”。实施麒沾马同城化建设三年行动计划，完成麒马大道等4条同城化主干道建设。继续开展城乡人居环境提升行动，改造提升寥廓公园、玉林公园等5个城市公园，推进149个城乡环境修整完善项目，完成南盘江综合治理一期工程和162个工程治理项目建设。深入开展“厕所革命”，确保县城建成区公厕数量提高到每平方公里5座以上。新开工棚户区改造4107套。组建曲靖文化研究院，开展城市经济发展课题研究，挖掘城市文化，提升城市软实力。创新体育赛事、产业论坛、商贸洽谈等活动载体，不断扩大城市影响力。

积极实施乡村振兴战略。积极构建农村现代产业体系，在稳定粮食生产能力的基础上，按照“科研+种植养殖+深加工+流通”的全产业链理念，推行“大产业+新主体+新平台”模式，推动马铃薯、生猪、蔬菜、蓝莓及猕猴桃等优势产业发展壮大。流转农村土地30万亩以上，组建10个农业产业化联合体，新增市级农民合作示范社18个、家庭农场180个、“三品一标”认证35个、云南名牌农产品2个以上。大力发展农产品精深加工，着力解决农产品加工业产值占比较低的问题。加强生态宜居乡村建设，强化乡村规划编制及巡察，科学引导民居建设，逐步解决“只见新房、不见新村”的问题。完成107个建制村环境综合整治任务，新增农村卫生厕所1000个以上。加强农村精神文明建设，深入推进科技、文化、人才、心理疏导“四下乡”活动，弘扬乡风正气。加强和创新乡村治理，以法治为保障、德治为基础、村民自治为根本，推动乡村治理“三治融合”。

大力发展文体旅产业。把文化产业发展放在更加重要的位置，启动印象曲靖、南中磐石等一批文化精品项目建设，确保每个县（市、区）至少新增2户规模以上文化企业。建设一批集观光、体验、科普为一体的田园综合体，推动城市农业与文化旅游深度融合。加快重点景区建设步伐，力争高科技数字影视产业园等项目开工建设。推进全域旅游示范区创建，举办旅游产品设计创意大赛，策划一批钻石旅游线路，加快罗平旅游度假区等20个项目建设，确保“一部手机游云南”与全省同步上线运行。完善体育赛事基础设施，建成国家高原体育综合赛训基地，高水平举办世界铁人三项赛等国际国内知名赛事。

（四）坚定不移深化改革扩大开放

深化重点领域改革。全面完成市级政府性投融资公司规范整合和转型升级、曲靖商业银行增资扩股和更名工作。理顺市区两级城市管理、经济管理、社会服务职能，构建权责对等、运行高效的城市管理体制。加大“放管服”改革力度，全面推行“一颗印章管审批”“互联网+政务服务+公共资源交易”，让群众和企业到政府办事最多跑一次。推进“证照分离”“证照同办”改革，逐步取消工业产品生产许可，推动市场准入类行政许可由一个部门集中办理。优化财政收入结构，确保税收收入占财政收入的70%以上，对非税收入超过50%的县（市、区）挂牌督办、限期整改。改革财政资金支持市场主体管理使用办法，确保支持条件、申请程序、使用情况全部公开。探索建立机构编制动态管理机制，适时调整优化机构编制资源。破除制约教育、卫生、养老、社会保障等民生事业发展的体制机制障碍，扩大优质资源覆盖面。深化广播电视媒体改革，组建曲靖广电传媒集团。

加大对外开放力度。加强对外交流合作，建设曲靖面向南亚东南亚经贸文化交流中心。发挥职业教育优势，加大劳务输出力度。大力发展外向型经济，吸引外资企业在曲靖投资兴业，鼓励民营企业积极嫁接外资。围绕主导产品培育自主出口品牌，力争外贸进出口总额达10亿美元。

（五）持续加强基础设施建设

加强综合交通基础设施建设。推动三宝至清水、召夸至泸西、富源至兴义、寻甸至沾益、罗平至八大河、宣威至威宁高速公路实质性开工建设，加快宣威至富源、麒麟至泸西、沪昆高速改扩容富源至沾益段高速公路前期工作。实施国道改造提升工程83公里。新建和改扩建“四好农村路”1000公里。配合做好渝昆高铁、南昆铁路扩能改造、师宗至文山铁路项目前期工作，开工建设宣威支线机场和会泽、罗平、沾益通用机场。

加强水利基础设施建设。扎实做好水库移民安置等基础性工作，加快阿岗、车马碧等36件在建骨干水源工程建设，新开工5件骨干水源工程、48件以上小（二）型病险水库除险加固工程，做好黑滩河水库前期工作。巩固提升饮水安全20万人。

加强能源基础设施建设。加快市应急气源储备中心建设，推进中缅天然气管道昭通支线曲靖段、陆良支线二期项目建设，确保中心城区管网入户率达35%以上。完成10千伏及以下配网项目523个，建成35千伏及以上输变电工程8个。

加强信息基础设施建设。争取国家“智慧城市”试点，建立“智慧城市”产业基金，完善项目建设运营管理模式，启动规划、旅游、食药监等“智慧曲靖”项目建设，力争建成云中心。实施高速公路、旅游景区4G网络全覆盖工程，扩大光纤宽带和4G通信网络自然村覆盖面。

加强物流基础设施建设。推进全国区域级流通节点城市建设，提升物流信息化水平，积极发展多式联运、高铁快运、智能仓储。加快2个物流园区、2个物流中心、5个大型交易市场建设，完善农产品冷链物流体系，改善城乡末端配送设施条件，构建三级物流体系和四级快递物流设施体系。

（六）全面提高民生保障水平

推进区域教育中心建设。新建幼儿园120所，确保普惠性幼儿园比例达80%以上。加强中小学标准化建设，新建中小学5所，完成“全面改薄”200所，创建“八优”学校100所。消除义务教育阶段66人以上超大班额，确保富源、陆良通过国家义务教育基本均衡发展评估认定。推动曲靖二中、曲靖民中创建省一级一等高中，确保优质普通高中覆盖面达60%以上。深化职业教育改革，力争曲靖职业技术学院一期建成招生。启动曲靖医专新校区建设，支持曲靖师范学院申报硕士学位授予点。鼓励社会力量兴办教育，规范中小学校外培训市场。

推进区域医疗卫生中心建设。加快推进市中医院新院等项目建设，确保市妇幼保健院分院投入使用，建成心血管疾病等5个特色专科诊疗中心，确保县乡紧密型医疗联合体全覆盖。深化医药卫生体制改革，推进分级诊疗等制度建设，提高家庭医生签约服务覆盖率，实施远程诊疗“乡乡通”工程。实施改善医疗服务3年行动计划，大力提升基层中医药服务能力。完善妇幼健康服务体系和计划生育服务管理。加强重大疾病预防控制。推进国家级医养结合试点，力争医康养大健康产业园落地。

增强社会保障能力。实施创业就业促进工程，扶持创业1.2万人，新增城镇就业4万人。实行企业（职业）年金制度，健全职工工资稳定增长机制，依法维护劳动者合法权益，构建和谐劳动关系。完善社会保障体系，提高城乡低保、特困供养标准和补助水平，确保基本社会保险参保覆盖率达95%以上。推进养老服务标准化建设，新增养老机构床位1070张。深化殡葬改革，确保火化率提高到80%以上。

加快文化事业发展。提升爱国主义教育基地服务功能，加强青少年思想道德建设。新建一批乡（镇、街道）文化站、基层综合文化服务中心、社区图书馆、乡贤书院，倡导全民阅读，完成国家公共文化服务体系示范区创建任务。加快农村公益电影固定放映点建设，启动本地广播电视节目全覆盖工程。

（七）加大生态文明建设力度

强化生态环境监管。严格落实生态环境保护责任，确保中央、省环保督察反馈问题有效整改。开展第二次全国污染源普查，编制曲靖市环境总体规划，抓好生态环境监管体制改革，理顺自然保护区管理体制。加强环境监测，加大环保执法力度，严惩破坏生态环境行为。

强化环境保护治理。深入开展蓝天保卫专项行动，加强大气污染防治，推进清洁生产，加快清洁能源替代利用，划定高污染燃料禁燃区，确保全市环境空气质量优良率稳中有升。深入开展碧水青山专项行动，全面落实河长制，加大河湖管理保护力度，强化饮用水全过程监测监管，确保重要江河水功能区水质达标率达87%以上，国家和省级考核断面水质达标率达81.8%。深入开展净土安居专项行动，建立土壤环境调查评估制度，严控工矿污染、农业污染、生活污染，推进污染地块土壤治理与修复，加大重金属污染治理和固体废弃物处置力度。

强化生态环境建设。深入推进国家森林城市创建，启动大规模国土绿化行动，加大高速公路、高铁沿线和城市面山生态修复力度，治理水土流失面积423万亩，完成营造林60万亩，确保森林覆盖率达48%。

（八）提高社会风险防范和化解能力

着力防范和化解金融风险。开展互联网金融风险专项整治，严厉打击恶意逃废债务、非法集资等行为，严守不发生区域性系统性金融风险的底线。明晰债务主体，坚持“谁使用、谁偿还”。积极争取地方政府专项债券支持，严格政府债务限额和预算管理，设置政府债务“天花板”。

着力防范和化解安全生产风险。启动国家安全发展示范城市创建，健全安全生产巡查机制，深入开展重点行业领域安全生产专项整治，坚决遏制重特大安全事故发生。推进标准化、机械化、信息化、智能化矿井建设，确保复产复建矿井实现信息化监管全覆盖。加快标准化农产品交易市场建设，确保国家食品安全示范城市创建通过评估验收。

着力防范和化解社会稳定风险。持续推进平安曲靖建设，完善信息化、立体化社会治安防控体系，深入开展扫黑除恶专项斗争，打好禁毒防艾人民战争。深化法治曲靖建设，扎实推进“七五”普法，探索建立媒体公益普法制度，依法推进社区矫正，提高全社会法治意识。加快法治信访进程，进一步推进诉访分离，完善矛盾纠纷多元化解机制，及时就地解决人民群众合理诉求。

开展第四次全国经济普查、第三次全国土地调查、第二次全国地名普查。落实党的民族宗教政策，深化民族团结进步示范区建设，发挥宗教界爱国人士、信教群众在促进经济社会发展中的积极作用。加强国防后备力量建设，做好双拥优抚安置、国防动员、民兵预备役和人民防空工作。统筹做好外事、侨务、地震、档案、保密、地方志、社会科学等工作，推进红十字、慈善等事业发展。

四、努力建设人民满意政府

各位代表，打铁必须自身硬。新时代新任务对政府治理能力和水平提出了新的更高要求，我们必须以习近平新时代中国特色社会主义思想武装头脑，牢固树立“四个意识”，聚精会神抓好政府自身建设，不断提升政府治理能力现代化水平，努力建设人民满意政府。

——建设服务型政府。自觉践行以人民为中心的发展思想，深入一线问政于民、问需于民、问计于民，集思广益、汇聚民智，做到决策部署依民而作、办法措施为民而定、工作成效由民而评。勇于跳出思维定势，克服工作惯性，摆脱路径依赖，牢固树立“特区”思维，增强“特区”意识，启动“最多跑一次”改革，依法依规、积极主动服务市场主体，着力构建“亲”“清”新型政商关系。

——建设效能型政府。落实首问负责制、限时办结制、服务承诺制、责任追究制，提高行政效率。全面推广使用政务督办APP，实现政务督办工作全过程在线管理，确保中央、省、市各项决策部署落地生根。建立重大决策实施情况动态调整和效果评估机制，加大治庸治懒治散力度，对执行不力、政令不畅、失职渎职等行为，严肃责任追究。

——建设法治型政府。严格落实法治政府建设主体责任，科学制定政府立法工作计划，健全行政规范性文件审查、备案、公布等制度，提高立法质量。完善重大行政决策的公众参与、专家论证、风险评估、合法性审查、集体讨论决定机制，推进行政决策科学化。全面推行法律顾问制度和公职律师制度。扎实推进政务公开，自觉接受人大及其常委会的法律监督、工作监督和人民政协的民主监督，认真办理人大代表议案、建议和政协提案，自觉接受民主党派监督、人民群众和社会舆论监督，推动审计监督全覆盖。畅通行政复议申请渠道，推动行政机关负责人出庭应诉，自觉履行人民法院生效判决。

——建设信用型政府。牢固树立“功成不必在我”的理念，坚持“一张蓝图干到底”，用实绩证明组织没选错人、人民没投错票。坚决抵制弄虚作假、形式主义等作风，坚决抵制工作中不顾客观条件、随意承诺等行为，减少行政行为的主观性、随意性，提高政府公信力。建立守信激励和失信惩戒制度，依托“信用曲靖”事中事后综合监管服务平台，加强社会对政府部门和公职人员的评价监督，发挥政府在社会信用体系建设中的表率作用。

——建设廉洁型政府。履行好政府党组管党治党责任，推进“两学一做”学习教育常态化制度化，深入开展“不忘初心、牢记使命”主题教育。严格落实“一岗双责”，坚持每月召开一次政府党组会议、研究一次党建及党风廉政建设工作，扎实抓好巡视巡察反馈问题整改。深入落实村级财务阳光监管、小微权力制约的各项措施，坚决遏制基层“微腐败”。习惯在受监督和约束的环境中工作生活，认真贯彻落实中央八项规定和实施细则精神以及省委、市委实施办法，密切关注享乐主义、奢靡之风新动向新表现，持之以恒正风肃纪，努力营造风清气正的政务环境。

各位代表，积力所举，无往不胜；众智所为，无所不成。让我们更加紧密地团结在以习近平同志为核心的党中央周围，在省委、省政府和市委的坚强领导下，紧紧依靠全市人民，不忘初心、牢记使命，振奋精神、实干兴市，为谱写新时代曲靖高质量跨越式发展新篇章而努力奋斗！
